# Stochastic Tunneling of Two Mutations in a Population of Cancer Cells

**DOI:** 10.1371/journal.pone.0065724

**Published:** 2013-06-26

**Authors:** Hiroshi Haeno, Yosef E. Maruvka, Yoh Iwasa, Franziska Michor

**Affiliations:** 1 Department of Biology, Faculty of Sciences, Kyushu University, Fukuoka, Japan; 2 Department of Biostatistics and Computational Biology, Dana-Farber Cancer Institute, and Department of Biostatistics, Harvard School of Public Health, Boston, Massachusetts, United States of America; Queen's University Belfast, United Kingdom

## Abstract

Cancer initiation, progression, and the emergence of drug resistance are driven by specific genetic and/or epigenetic alterations such as point mutations, structural alterations, DNA methylation and histone modification changes. These alterations may confer advantageous, deleterious or neutral effects to mutated cells. Previous studies showed that cells harboring two particular alterations may arise in a fixed-size population even in the absence of an intermediate state in which cells harboring only the first alteration take over the population; this phenomenon is called stochastic tunneling. Here, we investigated a stochastic Moran model in which two alterations emerge in a cell population of fixed size. We developed a novel approach to comprehensively describe the evolutionary dynamics of stochastic tunneling of two mutations. We considered the scenarios of large mutation rates and various fitness values and validated the accuracy of the mathematical predictions with exact stochastic computer simulations. Our theory is applicable to situations in which two alterations are accumulated in a fixed-size population of binary dividing cells.

## Introduction

Genetic and epigenetic alterations in signaling pathways, DNA repair mechanisms, the cell cycle, and apoptosis lead to abnormal reproduction, death, migration, genome stability, and other behaviors of cells, which may lead to the onset and progression of cancer [1]. For example, homozygous inactivation of the RB1 gene causes the childhood eye cancer retinoblastoma [2]. Similarly, a reciprocal translocation between chromosomes 9 and 22 leads to the creation of the BCR-ABL fusion oncoprotein resulting in chronic myeloid leukemia [3], [4]. Epigenetic alterations can also induce abnormalities in gene expression within cancer cells [5]. Furthermore, drug resistance in cancer cells is acquired by genetic and/or epigenetic changes: in the treatment of chronic myeloid leukemia, for instance, combination therapy of imatinib (Gleevec, STI571) and dasatinib (BMS-35482) often fails due to the emergence of only one or two genetic alterations within the tyrosine kinase domain of BCR-ABL [6].

While experimental studies have identified specific (epi)genetic changes and their consequences for cancer progression and drug resistance, mathematical investigations have provided insights into how tumor cells accumulate such alterations during tumorigenesis. In the 1950s, the multi-stage theory of carcinogenesis was proposed when Nordling, Armitage and Doll, and Fisher investigated the age distribution of cancer incidence with mathematical approaches [7], [8], [9]. In 1971, Knudson revealed, utilizing statistical analyses of the retinoblastoma incidence data, that two hits in an “anti-oncogene” are the rate-limiting steps in this disease [2]; this gene was later identified as the tumor suppressor RB1 [10]. In recent years, biological knowledge about population dynamics and molecular mechanisms of tumorigenesis, invasion, and therapeutic resistance have been incorporated into the mathematical models; for instance, tissue structures in particular cancer types [11], [12], [13], [14], [15], [16] and the evolution of drug resistance in cancer cells [17], [18], [19] were considered.

Much effort has been devoted to elucidating the dynamics of accumulating two (epi)genetic alterations in a population of a fixed number of cells. The theory that reveals the dynamics of accumulation of two specific mutations in a population is useful for predicting the risk of emergence and the rate of progression of cancer cells, and also for the kinetics of drug resistance. Moreover, the theory can be extended to more complicated cases in which more than two specific mutations play a role in malignant lesions. In 2003, Komarova et al. [20] derived analytic solutions of stochastic mutation-selection networks with an assumption that most of the time, the cell population is homogeneous with respect to relevant mutations. They defined stochastic tunneling as the case in which cells with two mutations appear from a lineage of cells harboring a single mutation; the latter eventually goes extinct instead of reaching fixation. They performed a precise analysis of the existence of stochastic tunnels and explicitly calculated the rate of tunneling [20]. In 2004, Nowak et al. [21] calculated the probability as function of time that at least one cell with two inactivated alleles of a tumor suppressor gene has been generated. They found three different kinetic laws: in small, intermediate, and large populations, it took, respectively, two, one, and zero rate-limiting steps to inactivate a tumor suppressor. They also studied the effect of chromosomal and other genetic instabilities. Small lesions without genetic instability required a very long time to inactivate the next TSG, whereas the same lesions with genetic instability posed a much greater risk for cancer progression [21]. Iwasa et al. [22], in the same year, derived the explicit tunneling rate for situations in which cells with one mutation were neutral or disadvantageous as compared to wild type cells, with cells with two mutations having the largest fitness. The analytical solutions provided an excellent fit to exact stochastic computer simulations [22]. In 2005, Weinreich and Chao [23] developed an analytical expression for the critical population size that defines the boundary between the regime of sequential fixation of two mutations and that of simultaneous fixation in a Wright-Fisher model; they also investigated the effect of recombination on this phenomenon [23]. In 2008, Schweinsberg investigated the waiting time for a large number of mutations to arise when the fitness change conferred by each mutation is negligible; ie. when the mutations are neutral [24]. Lynch studied the mean time to fixation of two mutations and the effects of recombination on this process in a large range of population sizes [25]. Weissman et al. [26] and Altland et al. [27] analyzed how recombination affects the expected time to achieve fixation of two mutations under the assumption that intermediate cell types are disadvantageous.

In 2009, Weissman et al. [28] calculated the rate of stochastic tunneling as a function of the mutation rates, the population size, and the fitness of the intermediate population harboring only a single mutation in the Wright-Fisher model. They found that when intermediate populations were close to neutral as compared to wild type cells, then stochastic tunneling easily emerged in large populations. In small populations, however, stochastic tunneling was much less likely to arise [28]. Later on, Proulx used elementary methods of analyzing stochastic processes to derive the probability of tunneling in the limit of large population sizes for both the Moran and Wright-Fisher models. He found that the probability of stochastic tunneling was twice as large in the Wright-Fisher model as in the Moran model [29].

Finally, diffusion approximations also represent a useful method for describing the evolutionary process of accumulating mutations in a large population of cells under the assumption of weak selection [30]. In 2009, Lehmann and Rousset [31] investigated multi-locus fixation probabilities under arbitrary strengths of selection in the Wright-Fisher model by using the tools of diffusion approximations. They showed that such fixation probabilities could be expressed in terms of selection coefficients weighted by the mean first passages times of ancestral gene lineages within a single ancestor. They then applied these results to investigate the Hill-Robertson Interference, i.e. stochastic tunneling of cell lineages [31].

Despite a wealth of forays into the dynamics of stochastic tunneling of two mutations within populations of cells, several critical questions remain. For instance, currently available approaches do not provide accurate predictions for situations in which mutation rates are large. Such scenarios, however, are important when considering mutation accumulation in cancer cells since many tumor types exhibit mutator phenotypes [32]–[37]. Furthermore, existing methods do not take into account all possible fitness effects of the individual cell types – such as increased fitness of cells with one mutation as compared to those with zero or two mutations.

In this paper, we addressed these scenarios to provide a general description of stochastic tunneling in a tumor cell population of constant size. Such a model describes many situations arising during tumorigenesis such as the dynamics of cancer initiation from a cellular compartment of a healthy tissue as well as the chronic phase of tumor progression [21], [38]. We designed three methods to calculate the probability of existence of a homogeneous population of cells, all of which harbor two mutations, at an arbitrary time point. One method demonstrated an accurate fit against all scenarios in numerical simulations, but had a large computational cost. The second method showed a very good fit with small computational cost; however, the predictions were not accurate in cases in which cells with two mutations had the same fitness as wild type cells. The last method produced accurate results in the latter situation of neutral fitness. By utilizing the best method for each parameter condition, we obtained an accurate approximation for the probability of a homogeneous population of cells with two mutations over time.

## Methods

### The mathematical model

Let us consider a population of *N* reproducing cells proliferating according to the Moran process [39]. One elementary time step of this process consists of a cell division and a cell death. For each division event, a cell is chosen at random proportional to fitness; the division event may produce a mutated daughter cell with a small probability. For each death event, one cell is chosen at random from the population. The total number of cells, *N*, is constant over time. These cells may accumulate (epi)genetic alterations and/or structural genomic changes; these are collectively referred to as “mutations”. We consider three types of cells: those harboring no mutations, denoted as type-0 cells, those harboring the first of a sequence of two mutations, denoted as type-1 cells, and those harboring both mutations, denoted as type-2 cells. Initially, the population consists entirely of type-0 cells; these cells have relative fitness (i.e. growth rate) 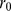. During each type-0 cell division, a type-1 cell may arise with probability equal to the mutation rate 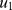. The fitness of type-1 cells is given by 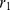. Finally, a type-2 cell may arise with probability 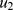 per type-1 cell division and has fitness 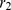. We assume that there is no back mutation because a mutation that exactly reverses the functional change caused by a specific mutation is rare compared to a mutation that causes a phenotypic change. Time is measured in units of cell divisions. Eventually, a type-2 cells will appear and may become dominant in the population; this event represents the evolution of adaptive cells.

**Figure 1:**
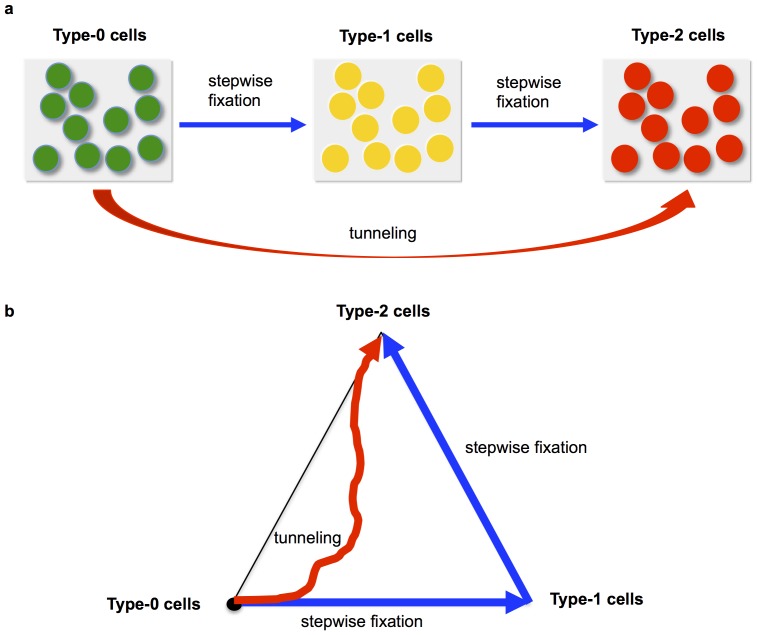
Schematic illustration of the model. Panel a shows the previously published approach to describing the evolutionary dynamics of two mutations in a fixed-size population of cells; only the transitions between homogeneous populations are considered. Panel b displays our novel approach, which encompasses considering the transitions in a heterogeneous population in detail.

In previous studies [20], [22], three states of a homogeneous population were considered: states in which all cells in the population are of type-0, type-1 or type-2 (**Figure 1a**). The authors then approximated the dynamics of fixation and tunneling in a heterogeneous population by using a fixation probability and a tunneling rate. This approximation, however, neglects the time from the appearance of a mutated cell to its fixation, as well as the effects of any additional mutational events during the time until fixation; this choice was made due to the observation that the waiting time of new mutation is usually much longer than the time of fixation in the parameter regimes considered. In some situations arising during tumorigenesis, however, these effects cannot be neglected – especially when mutation rates are large. In those cases, the previously derived approximation does not provide an accurate fit to the exact solution of the system. We thus aimed to consider the evolutionary dynamics of two mutations arising in a heterogeneous population using the methods described in the following (**Figure 1b**).

### Monte-Carlo simulations

We first performed Monte-Carlos simulations of the model describe above. Denote the number of type-0, type-1, and type-2 cells by *n*_0_, *n*_1_, and *n*_2_, respectively. Time is measured in cell cycles. During each time unit, one cell division and one cell death event occur to maintain a constant total number of cells. During one time step, the probability of a cell division of each cell type is given by
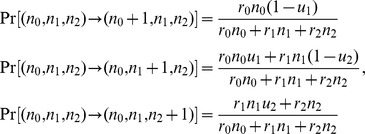
while the probability of a cell death of each cell type is given by
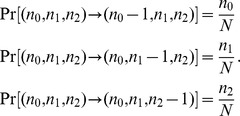



The initial condition is given by 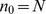 and 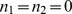. We performed 100,000 runs for each parameter set and obtained the fraction of cases in which the population consists entirely of type-2 cells at a given time.

### A novel approach

We extended our previously obtained results [22] to accurately describe situations in which mutation rates are large by considering the detailed transitions between states within a heterogeneous population. Denote by 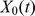, 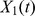, and 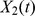, respectively, the probabilities at time *t* that the system consists exclusively of type-0, type-1, and type-2 cells. Then the dynamics of the population can be described by the forward Kolmogorov differential equations:
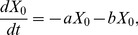
(1a)

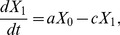
(1b)

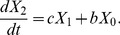
(1c)


The rate at which the population transitions from type-0 to type-1, *a*, is given by
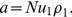
(2)Here 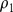 denotes the fixation probability of one type-1 cell in a population of *N*-1 type-0 cells and given by
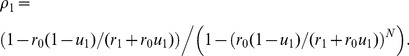
(3)


We have included the effect of the mutation rate in the fixation probability because, in situations when 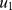 is very large, additional mutations can occur during the fixation of the former lineage. If 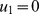, then 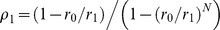, which was derived previously [20].

The tunneling rate, i.e. the rate at which the population transitions from type-0 to type-2 without the fixation of type-1 cells, *b*, is given by
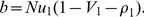
(4)Here 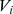 denotes the probability of non-appearance or extinction of a new type-2 lineage from *i* type-1 cells. With 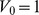 and 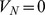, 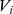 can numerically be calculated from the following equation:
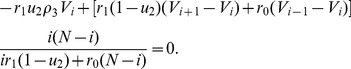
(5)Here 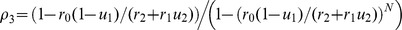. In both equations of 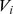 and 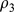, we include mutational events, which may increase or decrease the relative fitness of each cell type. See [22] for a detailed derivation of 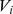.

Next, let us consider the following quantity:
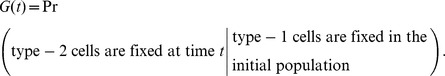
Then we have
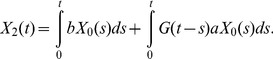
(6)If we assume
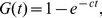
(7)where 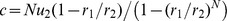, then we have
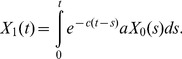
(8)By taking the derivative of Eq. (6) and (8), we obtain Eq. (1). Equation 1 no longer holds, however, when the second mutation rate, 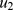, is very large since Equation 7 does not hold. Therefore, let us next calculate 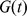 in a heterogeneous population of type-1 and type-2 cells.

Consider the *N*+1 states that are classified by the number of type-2 cells, *k = *0, 1, 2, …, *N*. Since we are interested in the situation after the emergence of type-1 cells, the number of type-1 cells becomes *N*-*k*. Then the transition probabilities are given by
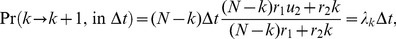
(9a)

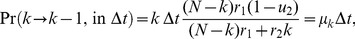
(9b)

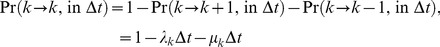
(9c)for *k = *1, 2, …, *N*-1. For *k = *0, we have 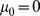. Note that the transition probability includes the second mutation rate, 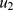, which is normally neglected when deriving the fixation probability in the Moran process due to the assumption of a very small mutation rate. Then we consider the following quantities:
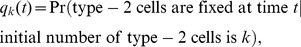
(10)where *k* = 0, 1, 2, …., *N*. Hence we have
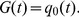
(11)By definition, we have the boundary condition, 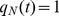, and the initial condition, 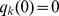 for *k* = 1, 2, 3, …, *N*-1. Then we obtain the following backward equation:
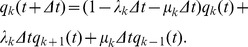
(12)By taking the limit when 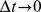, we have
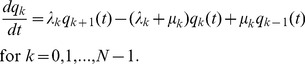
(13)Note that from Eq. (1a) and 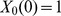, we have 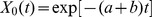. We set the second term of Eq. (6) as
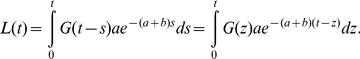
(14)Here 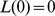 since 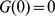. Finally, we have
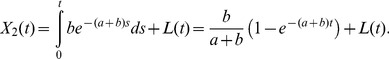
(15)By calculating the derivative of Eq. (14) we have
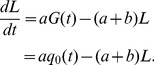
(16)
Eq. (15) provides good predictions for all ranges of mutation rates and relative fitness values of mutated cells, except when type-0 and type-2 cells are neutral (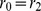) and the relative fitness of type-2 cells is smaller than that of type-0 cells (**Figure S2**). Although this method works in a wide parameter region, in order to investigate parameter regions where it does not accurately predict the exact dynamics, we consider two alternative methods.

### Systematic calculation of all transitions

Let us denote by 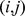 the state of the system in which the numbers of type-1 and type-2 cells are *i* and *j*, respectively. The state is confined within the following conditions: 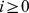, 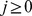, and 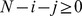. The system will eventually be absorbed into the state 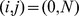, indicating that type-2 cells have reached fixation (i.e., 100% frequency) within the population. The fixation probability of type-2 cells from each state is then determined by using a backward calculation. For *i = *0, 1, 2, …, *N*, and *j = *0, 1, 2, 3,…, *N*, satisfying *i*+*j*≤*N*, we consider the probability, 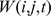, that type-2 cells have reached fixation before time *t*, starting from state 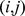. The boundary condition is given by
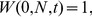
(17a)while the initial condition is given by
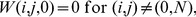
(17b)

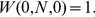
(17c)


Let us next consider the state transitions and derive the recurrence formulas for 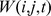. Within a short time interval, 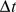, there exist six transitions:

[1] A transition from 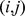 to 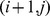 occurs when a type-0 cell dies and is replaced by a type-1 cell. There are two ways for this to occur: (i) a type-0 cell may die and a type-1 cell may divide (without mutating to give rise to a type-2 cell) or (ii) a type-0 cell may die and a type-0 cell may divide and mutate into a new type-1 cell. Then the transition probability is given by 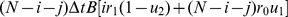. Here 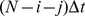 represents the probability of death of a type-0 cell during a short time interval, 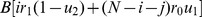 represents the probability of increasing the number of type-1 cells, and 
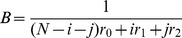
 gives the inverse of the total reaction rate.

[2] A transition from 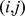 to 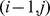 occurs when a type-1 cell dies and is replaced by a type-0 cell. The probability of this event is given by 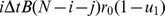.

[3] A transition from 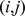 to 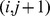 occurs when a type-0 cell dies and either a type-2 cell divides or a type-1 cell divides with a mutation, giving rise to a new type-2 cell. The transition probability of this event is given by 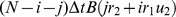.

[4] A transition from 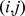 to 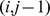 occurs when a type-2 cell dies and is replaced by type-0 cell. This probability is this event given by 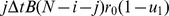.

[5] A transition from 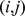 to 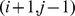 occurs when a type-2 cell dies and either a type-1 cell divides without a mutation or a type-0 cell divides with a mutation. The transition probability for this event is given by 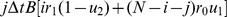.

[6] A transition from 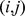 to 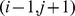 occurs when a type-1 cell dies and either a type-2 cell divides or a type-1 cell divides with a mutation. The transition probability for this event is given by 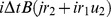.

Furthermore, there is a possibility that no transition occurs during a short time interval; the probability of no event occurring is given by one minus the sum of all the transition probabilities outlined above.

Considering these transitions between states, we have the following recurrence formula:
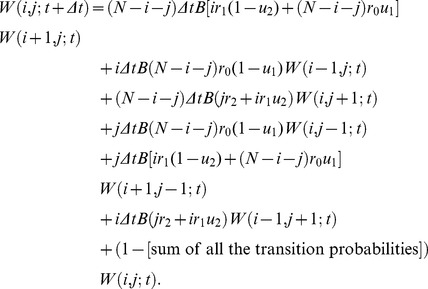
(18)


The left hand side of Eq. (18) denotes the fixation probability of a type-2 cell within the time interval Δ*t*, given that the initial state is 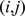. The right hand side is composed of the paths according to the type of event occurring during the time interval of length 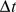. By calculating the limit when 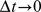, we have
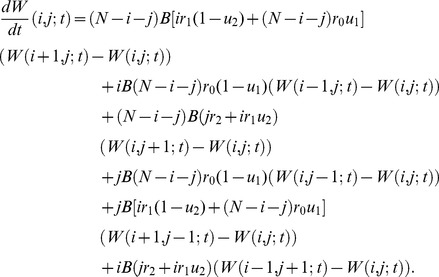
(19)


Using the initial condition Eq. (17b) and Eq. (17c), and the boundary condition Eq. (17a), we can numerically determine 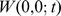, which represents the fixation probability of type-2 cells until time *t* in a population starting from *N* type-0 cells (**Figure S1**). Although this method provides accurate results, the time necessary for the numerical calculation, i.e. the number of equations, increases in a factorial way as the population size increases; on the other hand, it increases linearly in the first method. Therefore this method is not suitable for the determination of the dynamics in a large population.

### A simulation approach for the neutral case (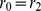)

An analytical formula describing the behavior of a system may serve several goals. One important goal is the ability to quickly obtain a prediction of the expected outcomes of a process, without the need for actually performing the process – no matter whether it is an experimental process or a Monte-Carlo simulation representing a large computational burden. This goal can be also achieved by approximating the time-consuming Monte-Caro simulation by another Monte-Carlo simulation that is much less computationally expensive. Even though the two simulations differ, the faster one may still serve as a good approximation of the slower one. Note that the use of the Wright-Fisher model in this context solely serves to increase the computational speed of our simulation, and is thus meant as an approximation to the Moran model. The Wright-Fisher model was *not* introduced to study an alternative population model, but instead was used as an approximation to the model under investigation (the Moran model) only.

Here we present the use of the tunneling process in the Wright-Fisher framework as an approximation for the tunneling process in the Moran framework. In the Moran framework, every generation is composed of *O*(*N*) random steps, while in the Wright-Fisher framework, the number of randomized steps per generation is independent of *N*. Instead, it depends only on the number of distinct cell types because there is a need only to generate the number of offspring each type will have in the next generation, and this can be done collectively.

We performed the Wright-Fisher Monte-Carlo simulation in the following way. At a given time *t* the state of the system is described by the vector ***n***(*t*), where *n*_0_ is the number of type-0 cells, *n*_1_ is the number of type-1 cells, and n_2_ is the number of type-2 cells. At every generation time step, the current population generates the next generation denoted by [*m*_0,_ *m*_1_,*m*_2_] from a multinomial distribution, with a probability vector 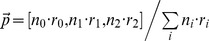. From the new offspring of type-0 cells, a binomially distributed number, with parameters *m*_0_ and *u*_1_, mutate and become type-1 cells, and from the offspring of type-1 cells, a binomially distributed number, with parameters *m*_1_ and *u*_2_, mutate and become type-2 cells. The process starts with *N*_0_ cells of type-0 and stops when one cell type reaches fixation or when the process reaches the maximal time. For a given set of parameter values, 100,000 replicates of the Monte-Carlo simulation were performed and the fixation probability was estimated as the fraction of cases in which type-2 cells reached fixation by time *t*. In order to compare the Wright-Fisher process to the Moran process, the population size *N*_0_ was then rescaled with the standard scaling of dividing by the standard deviation of the number of offspring each individual cell has, which is 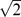 in the Moran process. Thus the population size used in the Wright-Fisher process is 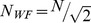.

Since the first method performs well for the non-neutral case, 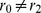, we applied the Wright-Fisher approximation only for the neutral case, 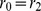. In general, the Wright-Fisher process has a similar fixation probability as the Moran process, and thus it can serve as a good approximation of the Moran model. In situations in which the fixation probability is very small, the difference between the two processes increases, thus rendering this approximation less exact; however, in these situations the approaches outlined above lead to accurate predictions.

## Results

**Figure 2:**
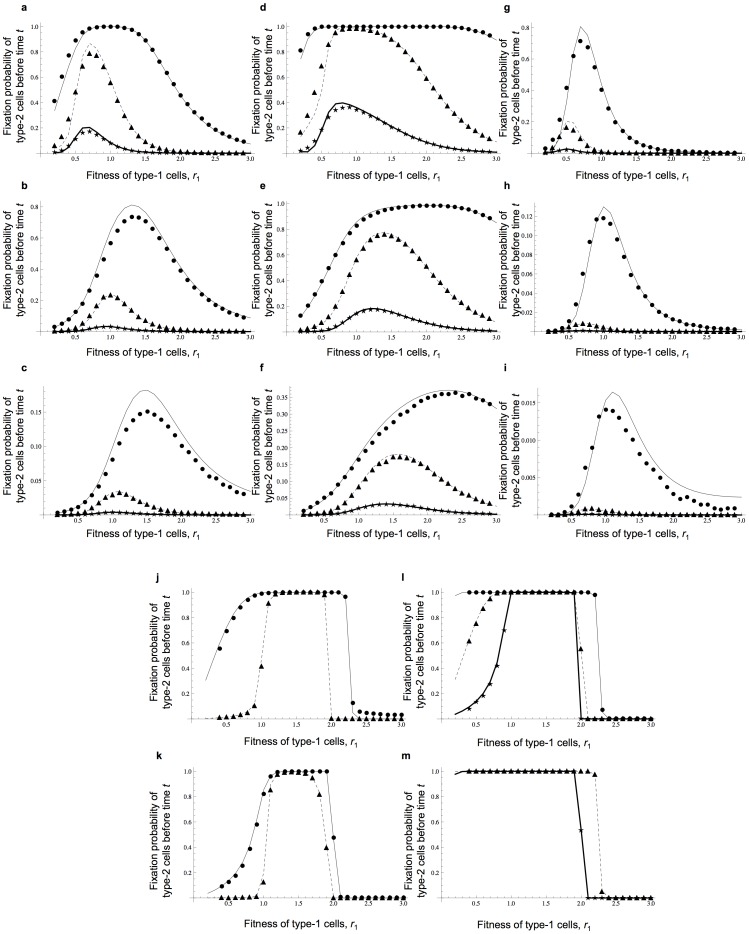
Results of our method. The figure shows the dependence of the probability that type-2 cells are fixed at time *t* on various parameters. Results by Eq. (15) are indicated by curves and those from direct computer simulations are shown by dots. The results of numerical calculations are connected and shown as a curve. Parameter values are 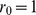, ; (a–i) 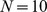 and 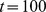; (a–c) 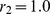; (d–f) 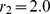; (g–i) 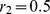; (a), (d), and (g) 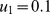; (b), (e), and (h) 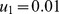; and (c), (f), and (i) 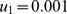. (a–i) Circles and thin curves represent 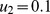, triangles and dotted lines represent 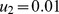, and stars and bold lines represent 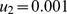. (j–m) 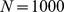, 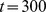 and 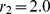; (j) circles and thin curves represent 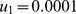 and 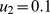, triangles and dotted lines represent 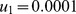 and 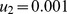; (k) circles and thin curves represent 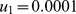 and 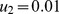, triangles and dotted lines represent 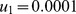 and 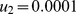; (l) circles and thin curves represent 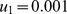 and 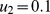, triangles and dotted lines represent 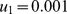 and 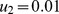, and stars and bold lines represent 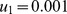 and 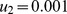; and (m) triangles and dotted lines represent 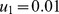 and 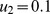, and stars and bold lines represent 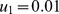 and 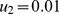.

We investigated the quality of fit of the approximations to the numerical results of the exact stochastic computer simulations. **Figure 2** displays the fit between the first approximation and Monte-Carlo simulation results in a wide parameter region (**Figure 2**). However, when the fitness value of type-2 cells is the same as that of type-0 cells, this approximation does not provide accurate predictions (**Figure S1**). We consider this parameter region in greater detail later. The comprehensive analysis showed that the probability of type-2 fixation increases when mutation rates are large and the fitness of type-2 cells is large.

Moreover, we found that there exists an optimal value of the fitness of type-1 cells that maximizes the fixation probability of type-2 cells at a given time point. If the fitness of type-2 cells is the same as that of type-0 cells and if the mutation rates are small, then the optimal value for the fitness of type-1 cells becomes 1 (**Figure 2c**). If the first mutation rate is very large, then a disadvantageous effect of the first mutation leads to the highest probability of type-2 fixation (**Figure 2a**). If the second mutation rate is very large, then an advantageous effect of the first mutation results in the highest probability of type-2 fixation (**Figure 2b–c**). If the fitness of type-2 cells is larger than that of type-0 cells, the optimal fitness of type-1 cells is between that of type-0 and type-2 cells in most cases (**Figure 2d–f**). However, when the first mutation rate is very large and the second mutation rate is very small, then a disadvantageous first mutation again leads to the highest probability of type-2 fixation (**Figure 2d**).

Furthermore, when the second mutation rate is very large and the first mutation rate is low, the optimal fitness of type-1 cells becomes even larger than that of type-2 cells (**Figure 2d–f**). Even though the fitness of type-2 cells is expected to be smaller than that of type-0 cells, fixation may still occur when the population size is small (**Figure 2g–i**). When type-2 cells are advantageous compared to type-0 cells, the tendency of the optimal fitness of type-1 cells does not depend on different values of the population size (**Figure 2j–m**). When time increases, then the fixation probability of population with two mutations also increases (data not shown).

**Figure 3:**
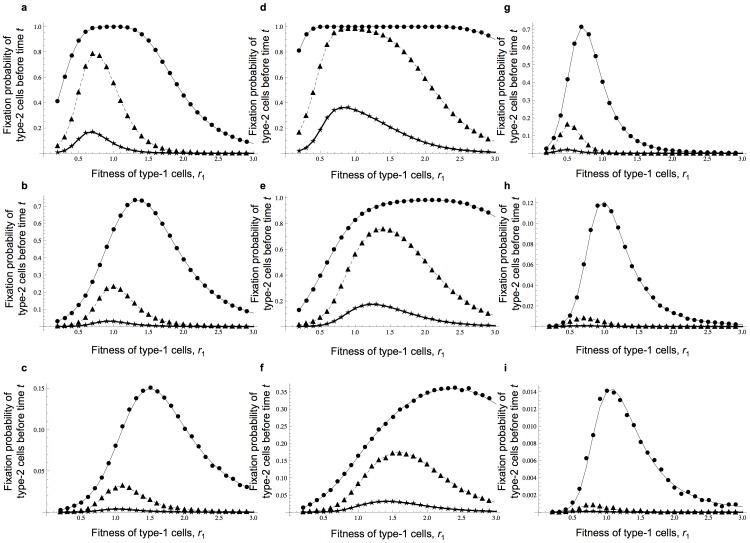
Precise predictions of the fixation probability of type-2 cells by systematic calculations of all transitions. The figure shows the dependence of the probability that type-2 cells are fixed at time *t* on various parameters. Results by systematic calculations, *W*(0,0,*t*), are indicated in curves and those from direct computer simulations are shown by dots. Parameter values are 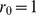 and 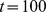; 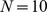; (a–c) 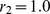; (d–f) 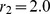; (g–i) 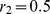; (a), (d), and (g) 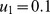; (b), (e), and (h) 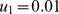; and (c), (f), and (i) 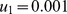. Circles and thin curves represent 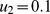, triangles and dotted lines represent 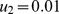, and stars and bold lines represent 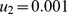.

We next investigated the predictions of the alternative method, which determines all transitions between states. Using the initial condition Eq. (17b) and Eq. (17c) and the boundary condition Eq. (17a), we numerically determined 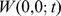, which represents the fixation probability of type-2 cells until time *t* in a population starting from *N* type-0 cells. **Figure 3** and **Figure S2** display the fit of 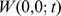 against results from direct computer simulations of the Moran model in a wide parameter region of small population sizes. The predictions provide an accurate fit to the simulation results.

**Figure 4:**
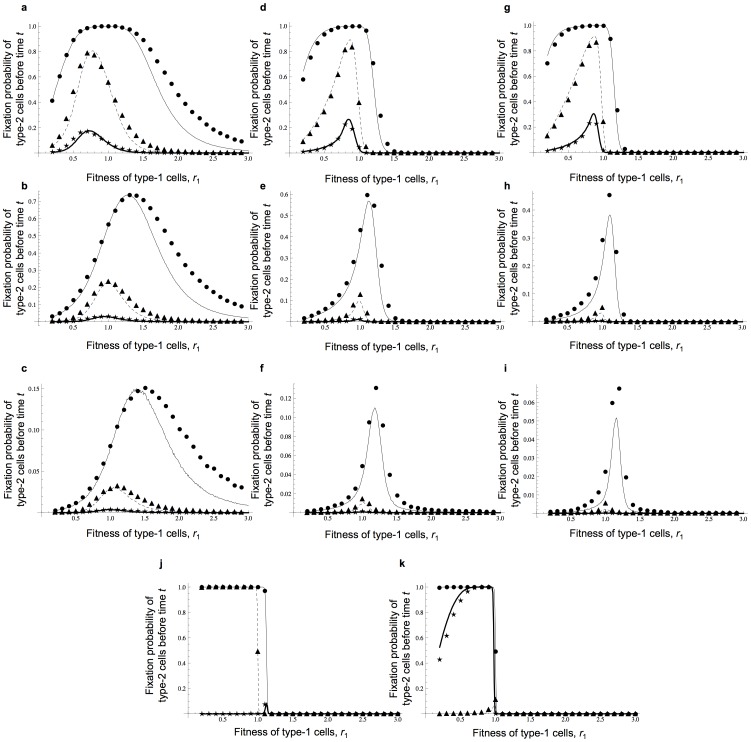
Results from a Wright-Fisher approximation. The figure shows the dependence of the probability that type-2 cells are fixed at time *t* on various parameters. Results by a Wright-Fisher framework are indicated by curves and those from direct computer simulations are shown by dots. Parameter values are 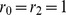 and 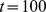; (a–c) 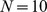; (d–f) 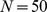; (g–i) 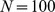; (a), (d), and (g) 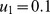; (b), (e), and (h) 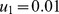; and (c), (f), and (i) 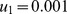. (a–i) Circles and thin curves represent 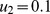, triangles and dotted lines represent 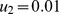, and stars and bold lines represent 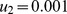. (j and k) 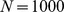; (j) circles and thin curves represent 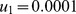 and 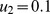, triangles and dotted lines represent 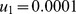 and 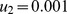; (k) circles and thin curves represent 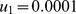 and 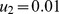, triangles and dotted lines represent 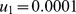 and 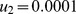.

Furthermore, we performed computational simulations using the Wright-Fisher framework to obtain the approximate results of Moran model (see alternative method 2 above). **Figure 4** displays the fit between the results of the Wright-Fisher model and those of the Moran model. This method provides accurate predictions for cases in which the fitness of type-2 cells is the same as the fitness of type-0 cells.

**Figure 5:**
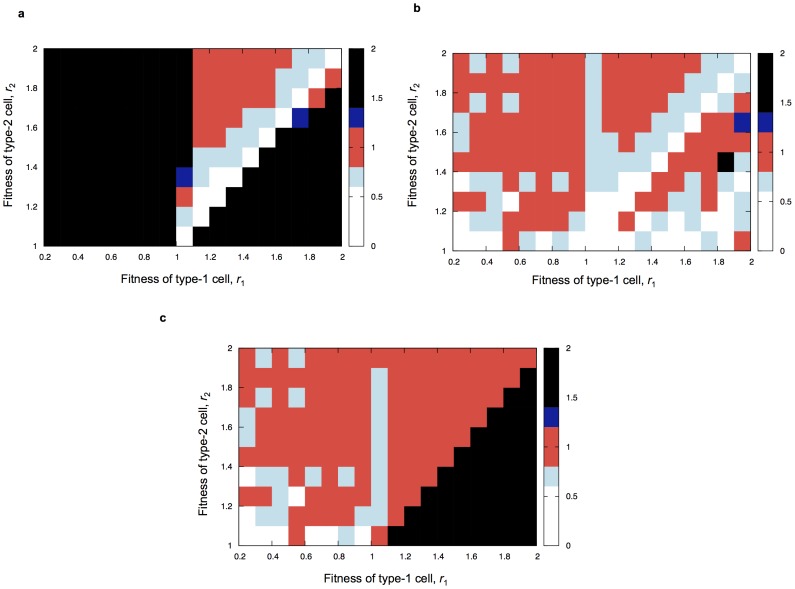
The region of tunneling. The figure shows a comparison between the simulation results and (panel a) Eq. (1) in reference [22] with *R = *0, (panel b) the tunneling formula in the previous paper (Eq. 1 in [22]), and (panel c) our new formula (Eq. 15). The color represents the fit between simulation results and each formula. The deviation is within 20% in a red region, from 20% to 40% overestimation in a light blue region, from 20% to 40% underestimation in a dark blue region, more than 40% overestimation in a white region, and more than 40% underestimation in a black region. Parameter values are 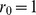, 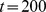, 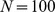, 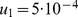 and 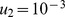.

We also investigated the parameter regimes in which the “stochastic tunneling” becomes important (**Figure 5**). Comparing the results by the direct simulation to forward Kolmogorov differential equation without tunneling term (Eq. 1 in [22] with *R* = 0), we found two parameter regions for stochastic tunneling (i) when the fitness of type-1 cells is smaller than that of type-0 cells and fitness of type-2 is the largest and (ii) when the fitness of type-1 cells is larger than that of type-0 and the fitness of type-2 cells is slightly smaller than that of type-1 cells (**Figure 5a**). In the figure, the non-red region represents bad fit (more than 20% overestimation or underestimation) of approximations against simulation results. We also showed the comparison between (i) the simulation results and tunneling formula in the previous paper (Eq. 1 in [22]), and (ii) the simulation results and our new formula (Eq. 15) (**Figure 5b–c**). In the region where 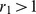 and 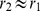, the new approximation works better than the previous formula, but when 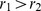 the new one underestimates the simulation results and the old one fits the simulation results more accurately.

**Figure 6:**
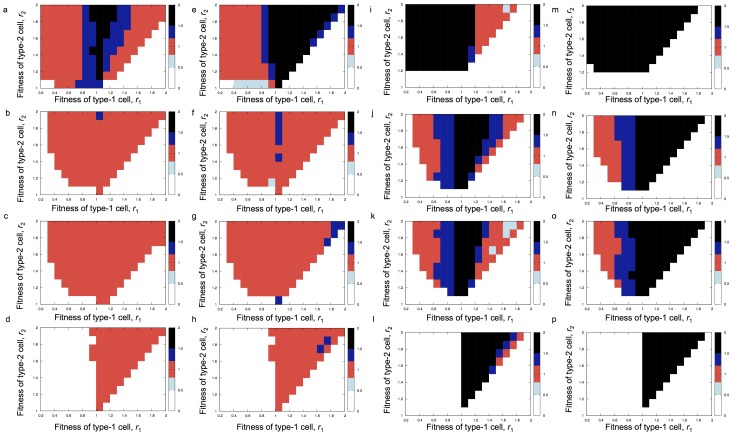
Predictions of the fixation probability of type-2 cells by different approaches. All predictions were divided by 0.5. When the ratio between prediction and 0.5 is 1, the color is red and signifies an accurate fit between the formula and the simulation result. When the ratio is much larger than 1, the color is blue and black and represents an overestimation of the formula. When the ratio is much smaller than 1, the color is light blue and signifies an underestimation of the formula. In the white region, we did not investigate the accuracy of the formulas because the time for the type-2 cell fixation became too long. The predictions by our approach (Eq. 15) are shown in panel a–d and i–l, and those by Iwasa et al. [22] are shown in panel e–h and m–p. Parameter values are 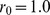, (a–h) 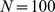; (i–p) 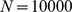; (a,e,i,m) 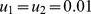; (b,f,j,n) 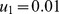, 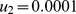; (c,g,k,o) 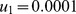, 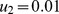; and (d,h,l,p) 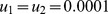. Time was chosen to obtain a probability of type-2 fixation of 0.5 for each parameter set.

Finally, we investigated the improvement of our new approximation over existing predictions of the fixation probability of type-2 cells at time *t* (**Figure 6**). First, from direct computer simulations and for each parameter set, we obtained the time at which the fixation probability of type-2 cells is 0.5. We then used these quantities for comparison with the predictions of existing approaches. **Figure 6** shows the predictions of our formula (Eq. 15) and that by Iwasa et al. [22] (Eq. 1 and Eq. 6). We found that, when the total number of cells was small (**Figure 6a–h**), both formulas showed good predictions except when both mutation rates were large (**Figure 6a and 6e**). In that case, our new formula worked better than the previous one [22]. When the total number of cells was large (**Figure 6i–p**), the previous formula did not work well. Also, when both fitness values of type-1 and type-2 were larger than 1, the new formula showed a better fit than the previous one.

We also investigated the accuracy of four other published approaches: those by Komarova et al. [20] (Section 3.2), Nowak et al. [21] (Eq. 6), Weissman et al. [28] (Eq. 25) and Proulx [29] (Eq. 11) (**Figure S3**). These four formulas did not exhibit as good a fit against the results obtained by direct computer simulations as our work. Since Komarova et al. [20] considered the probability of the first appearance of type-2 cells at time *t*, and not the fixation of type-2 cells, their equation always overestimates the probability of fixation (**Figure S3a–h**). The predictions by Nowak et al. [21] displayed a good fit in a certain parameter region (**Figure S3i–p**). Especially when the total cell number is large and the fitness of type-1 cells is smaller than that of type-0 cells, their predictions fit the results by direct computer simulations for a certain range of mutation rates. Overall, however, their predictions did not work well because they also did not consider the fixation of type-2 cells, but their appearance. Weissman et al. [28] performed a comprehensive study of the tunneling rate and the expected time until a mutant with *k* mutations appears in asexually reproducing populations. The case of *k* = 2 represents the same condition as in our current study. In their paper [28], the authors show the tunneling rate as Eq. 25; we included their tunneling rate in the tunneling term, *b*, in Eq. 1 in our formula (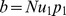, where *p*_1_ is given by Eq. 25 in [28]). We found that their approach did not provide a very accurate fit to results obtained from exact computer simulations (**Figure S3q–x**). Finally, the predictions by Proulx also did not exhibit a good fit (**Figure S3y–F**).

In summary, our new approach (Eq. 15) displayed the best fit against the direct computer simulations amongst all formulas investigated (**Figure 6**). However, we still need to perform systematic calculations of all transitions (Eq. 19) or direct computer simulations when the mutation rates and the population size are large and the fitness of type-1 is close to that of type-0 cells (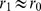) (**Figure 6a and i–l**). Moreover, the new formula does not work well when (i) type-1 cells are disadvantageous and the mutation rates and the population size are large (**Figure 6i**); and when (ii) type-1 cells are advantageous, mutation rates are small and the population size is large (**Figure 6l**). In such parameter regions, the systematic calculation of all transitions (Eq. 19) or direct computer simulations are necessary.

## Discussion

In this paper, we have performed a comprehensive analysis of the fixation probability of cells harboring two mutations; these mutations are accumulated sequentially in cells within a population of fixed size. Although the evolutionary dynamics of cells acquiring one or two mutations has been studied for decades, this work represents the first investigation of the fixation probabilities in the Moran model in a wide parameter region including large mutation rates and a disadvantageous fitness of cells harboring both mutations. A consideration of the risk of a cell population harboring two mutations, as well as the fixation probability of such cells, is important for situations arising during tumorigenesis such as the inactivation of tumor suppressor genes. When the time until fixation of type-2 cells is not negligible, the latter becomes more informative than the former. This situation occurs when the fitness of type-2 cells is not sufficiently advantageous or mutation rates are very large and it is not negligible [32], [33]. Our approach considers an approximation to the tunneling rate – the rate of transition from a population consisting entirely of type-0 cells to a population consisting entirely of type-2 cells, which represents an extension of our previous study [22]. This approach is computationally less expensive and provided good predictions for situations in which type-2 cells are advantageous as compared to type-0 cells. Note that we used large mutation rates in our analyses, at a range of 10^−4^ < *u* < 10^−1^; this choice was made since experimental evidence suggests that the mutation rate per base per cell division could increase up to these values due to phenomena such as chromosomal instability and microsatellite instability [33], [34].

We then investigated an approach considering all possible states of the population, consisting of all three cell types, and calculating all transitions among these states provided accurate predictions as tested by direct computer simulations. However, the time to calculate these predictions increases as the population size expands because the number of equations increases in a factorial way, and this method is thus infeasible to perform for large populations. For situations in which type-2 cells are neutral as compared to type-0 cells, we adopted the Wright-Fisher framework to obtain computationally faster approximations of the results from the Moran model because the first method does not provide accurate predictions in this parameter region.

These results are useful for considering the dynamics of mutation accumulation during cancer initiation, progression, and the emergence of resistance. A detailed kinetic understanding of the processes leading to cells that harbor a certain number of mutations can provide greater insights into tumorigenesis as well as allow predictions for the mutational composition of a tumor at certain time points. Furthermore, such a theory allows for a study of the circumstances that maximize the rate of evolution, i.e. the rate of mutation accumulation in cell populations. When investigating the optimum fitness of type-1 cells that maximizes the probability that a cell with two mutations has reached fixation within a population of cells, we found that, in a wide parameter region, the optimal fitness of type-1 cells is disadvantageous as compared to type-0 and type-2 cells when the first mutation rate is very large (**Figure 2a, d, g**). When the first mutation rate is large, then a large fraction of type-0 cell divisions contributes to an increase of type-1 cells by mutational events. This phenomenon could arise because once a mutated cell (according to our notation, a type-1 cell) appears, the clone it produces needs to undergo a large number of cell divisions to reach fixation (i.e. 100% frequency) in the population. This number of cell divisions equals at least the size of total population. During these cell divisions, the non-mutated cells (type-0 cells) experience a much larger number of cell divisions than type-1 cells because the initial number of type-0 cells is much larger than that of type-1 cells when the latter has just been produced. Then, during these cell divisions, additional mutations can emerge and will contribute to the increase of the number of type-1 cells.

Moreover several biological observations support the existence of this phenomenon. It is well known that genetic instability contributes to tumorigenesis; the rate of chromosomal loss or gain in genetically unstable cells has been measured to be about 0.01 per cell division [33]. Furthermore, mutations at different loci could result in the same phenotype of a new mutant because these mutations may affect the same signaling pathway in the cell [40], which leads to a high mutation rate for generating a particular phenotype. Finally, epigenetic changes may also occur at the same sites as genetic mutations, thus increasing the rates of alterations of that particular locus. Similarly to genetic instability, epigenetic instability can thus also lead to large mutation rates [41].

Therefore, the relative fitness of type-1 cells as compared to type-0 cells is effectively advantageous due to the high mutation rate, even though the numerical value of type-1 cell fitness is smaller than type-0 cell fitness. Once type-1 cells become dominant in the population, a small fitness value of type-1 cells maximizes the chance of type-2 cells to reach fixation in a population of type-1 cells. Moreover, when the second mutation rate is very large, the optimal fitness of type-1 cells is even larger than that of type-2 cells (**Figure 2d–f**). A large fitness value of type-1 cells enhances the increase of type-1 cells in a population of type-0 cells. In the process of reaching fixation of type-2 cells in a population of type-1 cells, a large mutation rate enhances the abundance of type-2 cells. These phenomena could arise due to the reasons described in the previous paragraph. However, the effects of additional mutations arising during the fixation process, i.e. while the cell population increases in abundance, are thus very important for the dynamics of mutation accumulation in a population of cells; such effects have not previously been described in detail.

Moreover, we investigated parameter regions in which “stochastic tunneling” occurs. In regimes in which 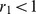 and 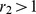, the equation without a tunneling term underestimates the simulation results (**Figure 5a**). Interestingly, we found that even when 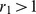 and 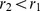, tunneling occurs, which had not been considered as being part of the tunneling regime previously. We confirmed that when 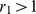 and 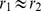, the new approximation provides more accurate predictions than the previous formulas, but when the fitness of type-2 is sufficiently smaller than that of type-1, the previous approach is better (**Figure 5b and c**).

In human tumors, the total number of cells is expected to be much larger than 1,000 cells, which is the parameter used for most of our studies. The reasons we considered relatively small population sizes are as follows: (i) Our model is not necessarily meant to consider only large, late-stage tumors with population sizes of the order of 10^9^ to 10^12^ cells. The model is thus designed to describe small, constant-size populations in which sequential mutations arise, and we describe the dynamics with which this process occurs. There are only few estimates for the population structure and cell numbers within healthy human tissues; for instance, a crypt in the human colon contains about 2,000 cells, which are replenished by a small number of stem cells (4–6) [42]. Since only the mutations arising in stem cells can be maintained indefinitely within the tissue without being “washed out” of the system by differentiation, it is the number of stem cells that represents the effective population size. (ii) In many tumor types, there exists a cellular differentiation hierarchy of tumor cells, subdividing the tumor into “cancer stem cells” and “cancer differentiated cells” [43]. It has been estimated that only about one in a million tumor cells are true stem cells in tumor types that adhere to this model [44]. Since only those tumor stem cells have unlimited self-renewal capacity, the accumulation of mutations needs to be considered in only this population to study the evolutionary dynamics of the entire tumor. For those reasons and for the computational speed of our analyses, we considered relatively small population sizes.

Our findings provide new insights into the evolutionary dynamics of cancer cells. We derived a theory of the accumulation of two mutations in a population of fixed size, and found that the frequency of mutational events determines the optimum fitness landscape for cancer cells in search of accumulating multiple mutations. Once plausible parameter values have been estimated, we are now able to obtain the fixation probability of population with two mutations at any time point. Particularly when mutations frequently occur in a cancer cell population, such as in the presence of genomic instability [32], [33], the effects of multiple mutational events during a short time interval need to be considered to obtain an accurate understanding of the dynamics of cancer cells. Although we considered a single intermediate population with one mutation, two mutations may cause two types of intermediate populations; models including such extensions will be considered in the future. Moreover, our work can be extended to investigate the emergence of a larger number of mutations, as well as a population subdivision into multiple compartments or niches; these niches may harbor cells that proliferate independently of cells in neighboring niches, or there may be migration of cells from one compartment to the next. In addition, we can consider time-dependent rates of mutation, cell division and death in addition to more complicated population structures. Such studies are ongoing and will provide further insight into the somatic evolution of cancer.

## Supporting Information

Figure S1
**Results** **of our approach.** The figure shows the dependence of the probability that type-2 cells are fixed in the population of cells at time *t* on various parameters. Results by Eq. (15) are indicated by curves and those from direct computer simulations are shown by dots. Parameter values are 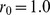 and 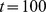; (a–g) 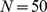; (h–m) 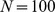; (a–c, h–j) 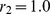; (d–f, k–m) 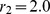; (g) 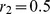; (a), (d), (g), (h), and (k) 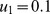; (b), (e), (i), and (l) 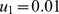; and (c), (f), (j), and (m) 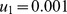. Circles and thin curves represent 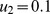, triangles and dotted lines represent 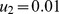, and stars and bold lines represent 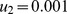.(TIFF)

Figure S2
**Precise predictions of the fixation probability of type-2 cells by systematic calculations of all transitions.** The figure shows the dependence of the probability that type-2 cells are fixed at time *t* on various parameters. Results by systematic calculations, *W*(0,0,*t*), are indicated by curves and those from direct computer simulations are shown by dots. Parameter values are 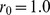 and 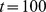; (a–g) 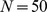; (h–m) 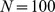; (a–c, h–j) 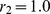; (d–f, k–m) 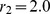; (g) 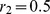; (a), (d), (g), (h), and (k) 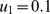; (b), (e), (i), and (l) 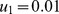; and (c), (f), (j), and (m) 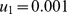. Circles and thin curves represent 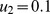, triangles and dotted lines represent 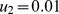, and stars and bold lines represent 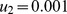.(TIFF)

Figure S3
**Predictions of the fixation probability of type-2 cells by different approaches.** This figure shows the results obtained using different approaches to calculate the fixation probability. The parameter values were chosen such that we obtained 0.5 for the fixation probability and the predictions by the formulas were divided by 0.5. When the ratio between prediction and 0.5 is 1, the color is red and signifies an accurate fit between the formula and the simulation result. When the ratio is much larger than 1, the color is blue and black and represents an overestimation of the formula. When the ratio is much smaller than 1, the color is light blue and signifies an underestimation of the formula. In the white region, we did not investigate the accuracy of the formulas because the time for the type-2 cell fixation became too long. In panels j, l, and p, when *r*_1_ is around 1.0, the predictions underestimate the simulation results and the white region between 0 and 0.1 appears. The predictions by the formula in Komarova et al. [20], in Nowak et al. [21], in Weissman et al. [28], and in Proulx [29] are shown in panel a–h in i–p, in q–x and in y-F, respectively. The color scheme in panel i–p was changed in order to be able to distinguish the underestimation by the formula from the low probability of fixation. Parameter values are 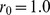, 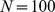 (a–d, i–l, q–t, y-B); 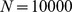 (e–h, m–p, u–x, C–F); 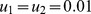 (a,e,i,m,q,u,y,C); 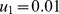, 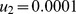;(b,f,j,n,r,v,z,D); 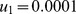, 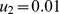 (c,g,k,o,s,w,A,E); and 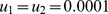 (d,h,l,p,t,x,B,F).(TIFF)
