# Supplementary figures and images for: Stochastic Tunneling of Two Mutations in a Population of Cancer Cells
Source: PLoS One. 2013 Jun 26;8(6):e65724. doi: 10.1371/journal.pone.0065724 (PMC3694076; doi:10.1371/journal.pone.0065724)

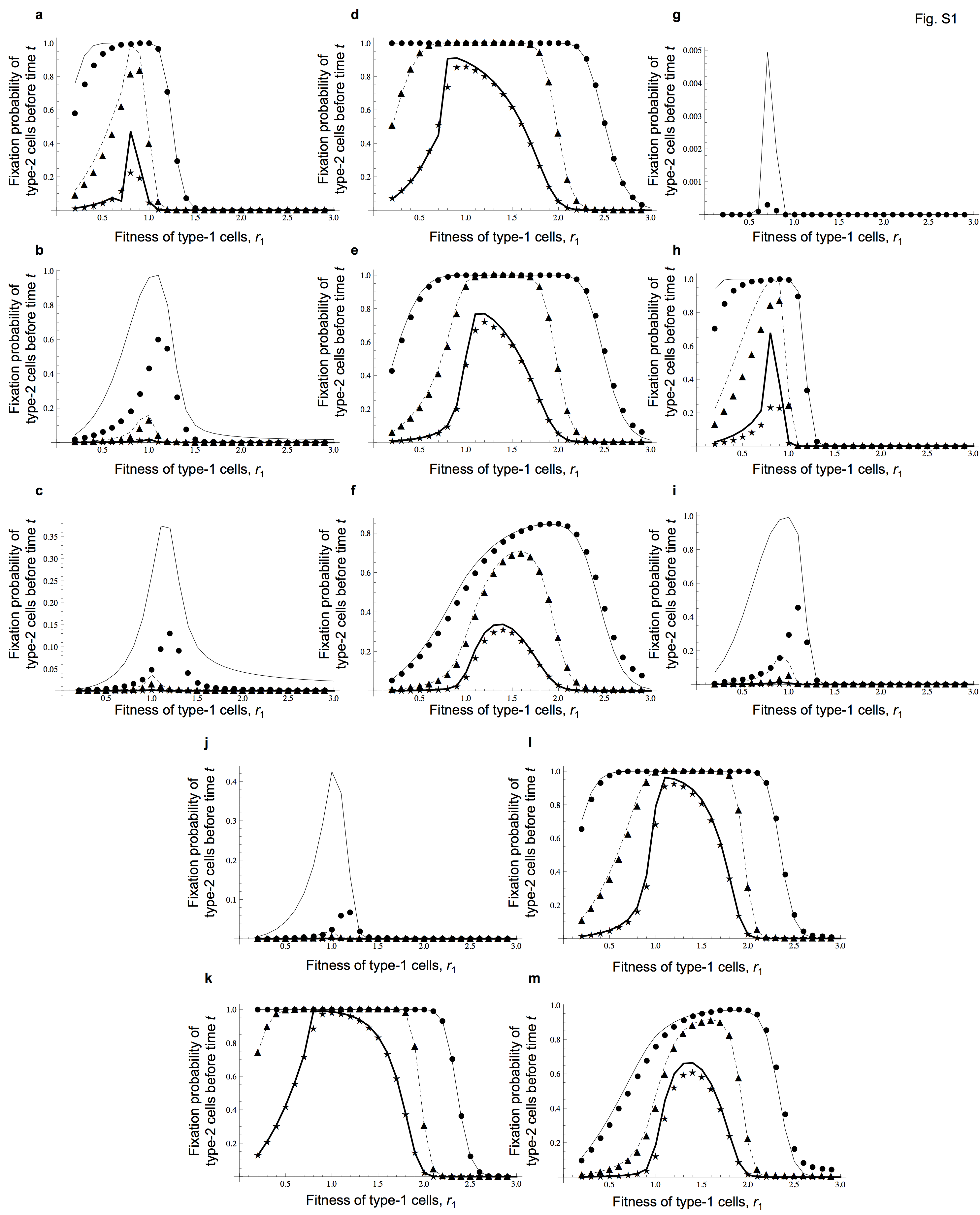

Supplement: Figure S1 — Results of our approach. The figure shows the dependence of the probability that type-2 cells are fixed in the population of cells at time t on various parameters. Results by Eq. (15) are indicated by curves and those from direct computer simulations are shown by dots. Parameter values are and ; (a–g) ; (h–m) ; (a–c, h–j) ; (d–f, k–m) ; (g) ; (a), (d), (g), (h), and (k) ; (b), (e), (i), and (l) ; and (c), (f), (j), and (m) . Circles and thin curves represent , triangles and dotted lines represent , and stars and bold lines represent . (TIFF) [file pone.0065724.s001.tiff]

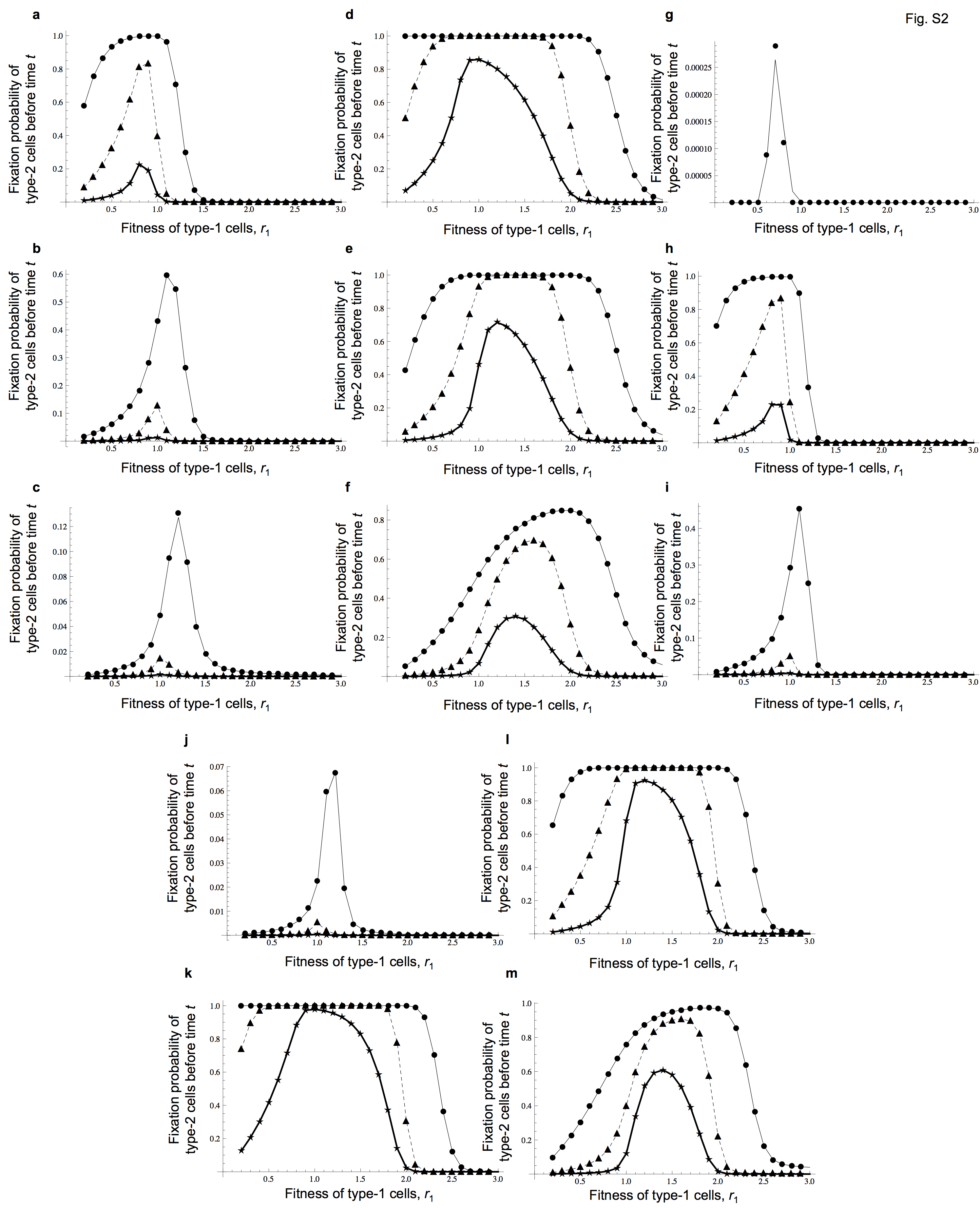

Supplement: Figure S2 — Precise predictions of the fixation probability of type-2 cells by systematic calculations of all transitions. The figure shows the dependence of the probability that type-2 cells are fixed at time t on various parameters. Results by systematic calculations, W(0,0,t), are indicated by curves and those from direct computer simulations are shown by dots. Parameter values are and ; (a–g) ; (h–m) ; (a–c, h–j) ; (d–f, k–m) ; (g) ; (a), (d), (g), (h), and (k) ; (b), (e), (i), and (l) ; and (c), (f), (j), and (m) . Circles and thin curves represent , triangles and dotted lines represent , and stars and bold lines represent . (TIFF) [file pone.0065724.s002.tiff]

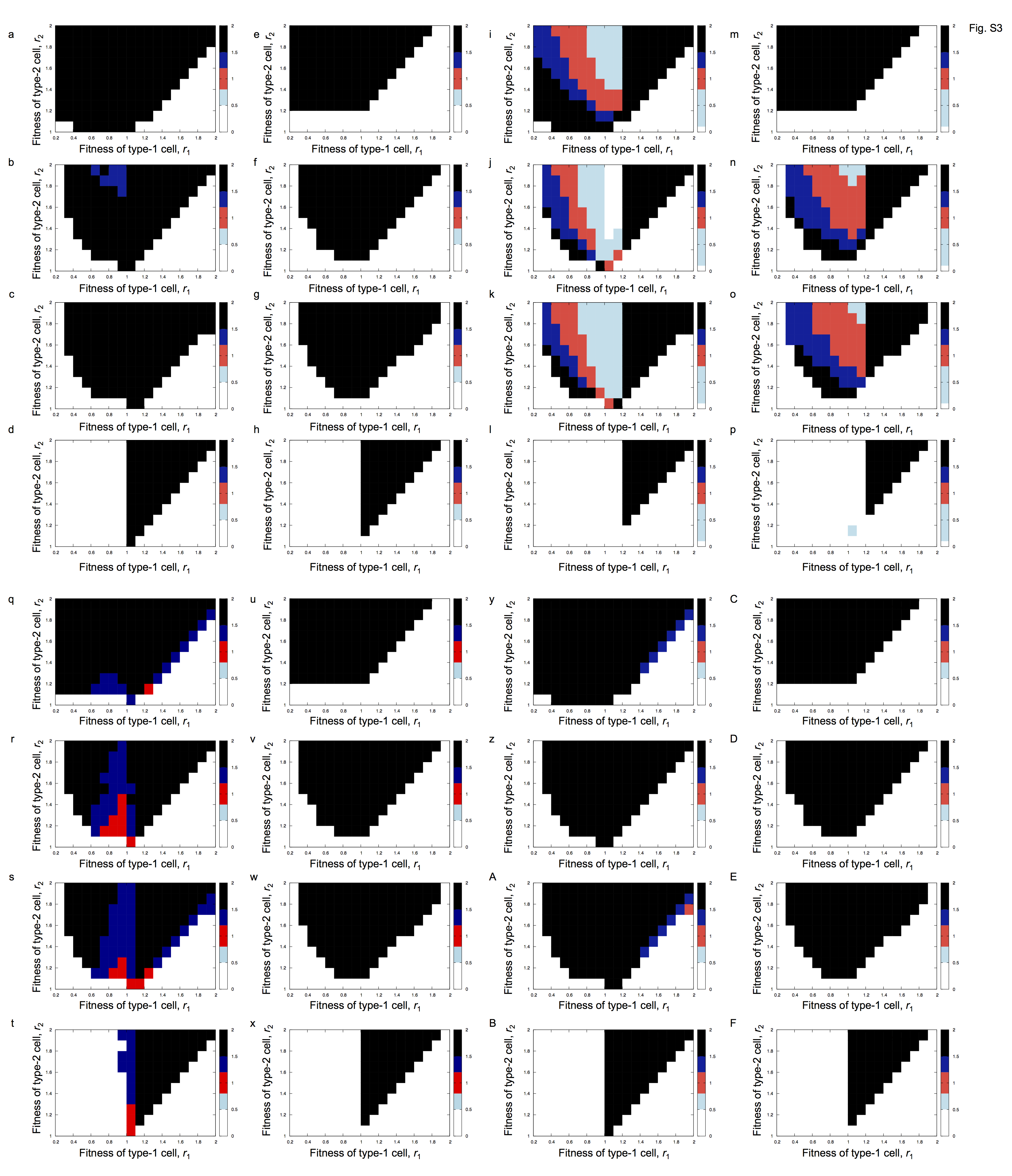

Supplement: Figure S3 — Predictions of the fixation probability of type-2 cells by different approaches. This figure shows the results obtained using different approaches to calculate the fixation probability. The parameter values were chosen such that we obtained 0.5 for the fixation probability and the predictions by the formulas were divided by 0.5. When the ratio between prediction and 0.5 is 1, the color is red and signifies an accurate fit between the formula and the simulation result. When the ratio is much larger than 1, the color is blue and black and represents an overestimation of the formula. When the ratio is much smaller than 1, the color is light blue and signifies an underestimation of the formula. In the white region, we did not investigate the accuracy of the formulas because the time for the type-2 cell fixation became too long. In panels j, l, and p, when r 1 is around 1.0, the predictions underestimate the simulation results and the white region between 0 and 0.1 appears. The predictions by the formula in Komarova et al. [20], in Nowak et al. [21], in Weissman et al. [28], and in Proulx [29] are shown in panel a–h in i–p, in q–x and in y-F, respectively. The color scheme in panel i–p was changed in order to be able to distinguish the underestimation by the formula from the low probability of fixation. Parameter values are , (a–d, i–l, q–t, y-B); (e–h, m–p, u–x, C–F); (a,e,i,m,q,u,y,C); , ;(b,f,j,n,r,v,z,D); , (c,g,k,o,s,w,A,E); and (d,h,l,p,t,x,B,F). (TIFF) [file pone.0065724.s003.tiff]
